# Genotype–phenotype correlations and protein domain-level predictors of cerebrovascular malformations in hereditary hemorrhagic telangiectasia

**DOI:** 10.1007/s00415-026-13777-2

**Published:** 2026-03-31

**Authors:** Carmelo Lucio Sturiale, Federico Cocilovo, Gianluca Trevisi, Matteo Palermo, Emanuela Lucci Cordisco, Luigi Di Martino, Elena Sonnini, Alessio Albanese, Francesco Doglietto, Roberto Pola, Eleonora Gaetani

**Affiliations:** 1 Department of Neurosurgery, Institute of Neurosurgery, Fondazione Policlinico Universitario A. Gemelli IRCCS, Università Cattolica del Sacro Cuore, L.go A. Gemelli 8, 00168 Rome, Italy; 2 Department of Neurosciences, Imaging and Clinical Sciences, G. D’Annunzio University, Chieti-Pescara, Italy; 3 UOC Genetica MedicaDipartimento di Scienze della Vita e Sanità Pubblica, Fondazione Policlinico Universitario A. Gemelli IRCCS, Università Cattolica del Sacro Cuore, Rome, Italy; 4 Department of Translational Medicine and Surgery, Fondazione Policlinico Universitario A. Gemelli IRCCS Università Cattolica del Sacro Cuore, 00168 Rome, Italy; 5 Unit of Internal Medicine, Cristo Re Hospital, 00167 Rome, Italy; 6 Department of Aging, Orthopedic, and Rheumatologic Sciences, Fondazione Policlinico Universitario A. Gemelli IRCCS, Università Cattolica del Sacro Cuore, 00168 Rome, Italy

**Keywords:** brain, arteriovenous malformation, HHT, genotype

## Abstract

**Background:**

Hereditary Hemorrhagic Telangiectasia (HHT) exhibits marked phenotypic heterogeneity. Although gene–organ associations are well established for visceral involvement, predictors of cerebrovascular malformations (CVMs), particularly brain arteriovenous malformations (bAVMs), remain incompletely defined. This study aimed to investigate genotype–phenotype correlations and identify predictors of bAVMs in a genetically confirmed HHT cohort.

**Methods:**

We conducted a retrospective analysis of 142 Caucasian patients with genetically confirmed HHT. Clinical manifestations were systematically assessed and correlated with the mutated gene (ENG, ACVRL1, and SMAD4), variant type (truncating vs. non-truncating), and protein domain location. Multivariable logistic regression was performed to identify independent predictors of bAVMs.

**Results:**

The cohort included 83 (58.5%) ACVRL1 and 53 (37.3%) ENG mutation carriers. Bivariate analysis demonstrated distinct phenotypic patterns. ENG mutations were strongly associated with pulmonary AVMs (*p* < 0.001) and bAVMs (*p* < 0.001), with bAVMs observed in 35.8% of ENG carriers compared with 3.6% of ACVRL1 carriers. In contrast, hepatic AVMs were more frequent among ACVRL1 carriers (44.6%), although this did not reach statistical significance (*p* = 0.086). In the multivariable logistic regression model (overall *p* < 0.001), younger age emerged as the sole independent predictor of bAVMs (OR 0.968, *p* = 0.040), whereas the mutated gene did not retain independent significance.

**Conclusion:**

ENG mutation carriers display a markedly increased cerebrovascular burden, confirming a gene-specific susceptibility to bAVMs. Younger age independently predicts bAVM presence. These findings support age- and genotype-informed risk stratification and may help refine screening strategies in HHT patients.

**Supplementary Information:**

The online version contains supplementary material available at 10.1007/s00415-026-13777-2.

## Introduction

Hereditary Hemorrhagic Telangiectasia (HHT), also known as Rendu–Osler–Weber syndrome, is an autosomal dominant vascular disorder characterized by mucocutaneous telangiectasias and visceral arteriovenous malformations (AVMs) [1, 2]. Its clinical expression is highly heterogeneous, ranging from recurrent epistaxis to life-threatening complications, such as paradoxical embolism through pulmonary shunts, stroke, and visceral hemorrhages. Pathogenic variants are represented by ENG, ACVRL1, and SMAD4 and account for most genetically confirmed cases. These mutations commonly lead to the disruption of the TGF-β/BMP signaling pathway, with consequent aberrant angiogenesis [3].

The prevalence of HHT is estimated approximately 1 in 5000 individuals, although higher rates have been reported in specific populations. The distribution of ACVRL1, ENG, and SMAD4 variants in our cohort reflects the large European and North American registries, consistent with the regional variability described by Sánchez et al. in 2020 [4].

Although classical gene–phenotype associations have been described, including the predominance of pulmonary AVMs (pAVMs) in ENG mutation carriers and hepatic AVMs (hAVMs) in ACVRL1 mutation carriers, significant inter-individual variability persists especially for the incidence of the different cerebrovascular phenotypes [5, 6].

On the other hand, the possibility of a predictive stratification of cerebrovascular risk based on the different genotypes nowadays represents a clinical priority, given the impact of early detection and targeted screening on morbidity.

Several studies have proposed that both variant type and protein-domain localization may influence vascular phenotype severity, although current evidence remains fragmentary and sometimes conflicting. Moreover, the contribution on the natural history of different variables, such as demographics, family clustering, and patients age at diagnosis, still requires clarification in order to plan surveillance strategies 

[7–11].

This study aims to characterize genotype–phenotype correlations in a cohort of Caucasian patients with HHT consecutively admitted to our tertiary referral center for HHT diagnosis and treatment at Fondazione Policlinico Universitario Agostino Gemelli IRCCS in Rome (Italy) between 2015 and 2025, with specific focus on cerebrovascular malformations. In addition, the study evaluates whether variant functional class and protein-domain localization provide incremental discriminatory value beyond the mutated gene itself and investigates independent predictors of brain AVMs (bAVMs) using multivariate modeling.

## Materials and methods

### Study design and patient cohort

This study was a retrospective analysis of a cohort of 142 patients with a confirmed diagnosis of Hereditary Hemorrhagic Telangiectasia (HHT) admitted to our tertiary referral center for HHT between 2015 and 2025. Patients were identified and data were collected from a dedicated HHT patient registry. All patients included in the study were of Caucasian descent. For each patient, we collected demographic information (age and sex), family history, and comprehensive clinical data regarding HHT-related manifestations. The study was conducted in accordance with institutional ethical guidelines.

### Genetic analysis

Genetic testing was performed in all 142 patients as part of the standardized diagnostic work‑up and represented an inclusion criterion for this study. Molecular analysis was carried out at our Medical Genetics Unit using validated diagnostic protocols for HHT. Genomic DNA was extracted from peripheral blood leukocytes, and the coding exons and exon–intron boundaries of ENG, ACVRL1, and SMAD4 were analyzed through next‑generation sequencing panels dedicated to hereditary vascular disorders. All pathogenic and likely pathogenic variants identified through NGS were subsequently confirmed by Sanger sequencing. Variant classification followed ACMG/AMP guidelines, and each mutation was annotated according to its predicted functional effect (truncating vs non‑truncating) and its protein domain localization (extracellular, intracellular, and untranslated). Only patients with a genetically confirmed diagnosis were included in the final cohort.

### Clinical data collection

The diagnosis of HHT was established according to the Curaçao criteria, which include epistaxis, telangiectasias, visceral involvement, and family history. A score of 3 out of 4 indicates a definite diagnosis of HHT, whereas fulfillment of 2 out of 4 criteria is considered suggestive of possible HHT. Each involvement of other organs was evaluated by reviewing radiological reports from diagnostic or screening imaging (CT, MRI, and US). We investigated the presence of pulmonary, hepatic, and cerebral arteriovenous malformations (AVMs), along with cerebral cavernomas, aneurysms, arteriovenous fistulas (AVFs), and developmental venous anomalies (DVAs). Additionally, we recorded stroke events and cerebral abscesses. All 142 patients underwent a complete and standardized diagnostic work‑up at our HHT referral center. Neuroimaging was performed in 100% of cases through brain MRI to assess brain AVMs and other cerebrovascular lesions. Moreover, all patients received comprehensive splanchnic screening, including total‑body contrast‑enhanced CT, abdominal ultrasound, and, when required, selective splanchnic or cerebral angiography to detect the presence of arteriovenous malformations in other organs. In addition, every patient was forwarded in a multidisciplinary follow-up pathway, including otolaryngology, cardiology, gastroenterology, internal medicine, neurosurgery, interventional radiology and neuroradiology, and vascular medicine consultations, in dedicated weekly HHT board sessions.

Because this study was designed as a cross‑sectional retrospective analysis, vascular malformations and cerebrovascular events were recorded if documented at any point in the patient’s medical history, whether at baseline or during subsequent evaluations. A uniform follow‑up duration was not available, as imaging and clinical assessments were performed according to individual clinical indications rather than predefined intervals.

### Statistical analysis

Descriptive statistics were employed to describe the cohort, including frequencies (%) for categorical variables and means with standard deviations for continuous variables.

The Chi-squared (*χ*^2^) test was used to investigate genotype–phenotype correlations. Specifically, we examined the associations between the mutated gene or the protein’s cellular allocation and the presence of vascular malformations. Additionally, we specifically analyzed the association between the mutated gene and the occurrence of acute cerebrovascular events (ischemic stroke and intracranial hemorrhage).

To identify independent predictors of bAVMs, a multivariable logistic regression model was constructed. The model included the mutated gene, patient sex, and mutation type as categorical factors, with patient age and Curaçao diagnostic score as continuous covariates. A generalized linear mixed model (GLMM) with 'Family' included as a random effect was initially considered to account for potential clustering of phenotypes within families. However, this model failed to converge due to the large proportion of families with only a single individual in the dataset, making the random-effects structure unstable. Therefore, a standard multivariable logistic regression was employed.

Finally, to evaluate the specificity of the identified risk factors, the same multivariable logistic regression framework (incorporating age, sex, mutated gene, and Curaçao score) was applied to predict the presence of other vascular phenotypes, including pulmonary and hepatic AVMs, as well as other cerebrovascular lesions (cavernomas, DVAs, dAVFs, and intracranial aneurysms).

All statistical analyses were performed using JASP software (version 0.95.3—Sep 30th, 2025). A two-sided p value of less than 0.05 was considered to indicate statistical significance.

## Results

### Patient cohort characteristics

Table 1 summarizes demographics, genetic and clinical characteristics of the study cohort.

**Table 1:** Demographic and clinical characteristics of the patient cohort stratified by genetic profile

Characteristic	ACVRL1 mutation (*n* = 83)	ENG mutation (*n* = 53)	SMAD4 mutation (*n* = 6)
*Demographics*			
Age (years)	52.2 ± 17.6	48.4 ± 17.9	41.2 ± 13
Female Sex	41 (49.4%)	26 (49.1%)	5 (83.3%)
Positive family history	83 (100%)	47 (88.7%)	3 (50%)
*Clinical manifestations*			
Epistaxis	80 (96.4%)	51 (96.2%)	5 (83.3%)
Cutaneous telangiectasias	74 (89.2%)	40 (75.5%)	4 (66.7%)
Gastrointestinal bleeding	15 (18.1%)	11 (20.8%)	1 (16.7%)
*Organ involvement*			
Pulmonary AVMs	17 (20.5%)	32 (60.4%)	1 (16.7%)
Hepatic AVMs	37 (44.6%)	14 (26.4%)	3 (50%)
Brain AVMs	3 (3.6%)	19 (35.8%)	1 (16.7%)
Cerebral cavernomas	2 (2.4%)	0 (0%)	2 (33.3%)
Brain aneurysms	4 (4.8%)	0 (0%)	0 (0%)
Brain dAVFs	1 (1.2%)	0 (0%)	0 (0%)
Brain DVAs	5 (6%)	1 (1.9%)	0 (0%)
Brain capillary telangiectasia	2 (2.4%)	1 (1.9%)	0 (0%)
Stroke	5 (6%)	9 (17%)	1 (16.7%)
Cerebral abscess	1 (1.2%)	3 (5.7%)	1 (16.7%)

Figures are expressed as *n* (%) or mean ± SD

The study cohort comprised 142 Caucasian patients, from 112 different families, nearly equally distributed by sex: 72 females (50.7%) and 70 males (49.3%). Mean age was 50.3 ± 17.65 years, with ages ranging from 10 to 82 years. Diagnosis of HHT was made according to the Curaçao criteria. One hundred-thirty-three patients had a positive family history of HHT (93.7%).

### Genetic profile

Genetic analysis of associated-HHT genes revealed that ACVRL1 gene mutations were the most common (83 patients, 58.5%), followed by mutations in the ENG gene (53 patients, 37.3%), while SMAD4 mutations were only present in 6 patients (4.2%).

Analysis of specific variants highlighted recurrent mutations: the most common was the c.1120C>T (p.Arg374Trp) in the ACVRL1 gene, identified in 12 individuals (8.5%).

To better characterize the cohort’s genotype, variants were classified based on their functional impact. Truncating variants were identified in 73 patients (51.4%). This group encompassed nonsense mutations (*n* = 28, 19.7%), frameshift variants resulting from deletions (*n* = 25, 17.6%) or insertions (*n* = 5, 3.5%), and splice-site defects (*n* = 7, 4.9%). Conversely, non-truncating variants accounted for the remaining 69 patients (48.6%). This latter group was predominantly characterized by missense mutations, which represented the most frequent mutation type in the entire cohort (*n* = 59, 41.5%).

### Clinical manifestations and organ involvement

Speaking of phenotypes, most patients presented with classical HHT signs. Epistaxis was reported in nearly all patients (136/141, 96.5%), followed by cutaneous telangiectasias (118, 83.1%) and gastrointestinal bleeds (27, 19.0%).

Additionally, organ-level analysis for detecting AVMs revealed significant involvement. Of note, 50 patients had pAVMs (35.2%), while 54 had hAVMs (38.0%).

Analysis of cerebral involvement found 23 patients presenting with bAVMs (16.2%), 4 patients with cerebral cavernomas (2.8%), 1 patient with dural arteriovenous fistula (dAVFs, 0.7%), 6 patients with developmental venous anomalies (DVA, 4.2%), 3 with brain capillary telangiectasias (2.1%), and 4 with cerebral distant aneurysms (2.8%). Cumulatively, strokes were seen in 19 patients (13.5%). Ischemic strokes were reported in 15 patients (10.6%) and hemorrhagic strokes in 7 (4.9%), with 3 of these patients (2.1%) showing a combination of hemorrhagic and ischemic stroke. Five patients developed cerebral abscesses (3.5%).

### Bivariate genotype–phenotype correlations

The initial statistical analysis assessed the associations between clinical phenotypes and mutated genes (Table 2**)**. A statistically significant association was found between the mutated gene and the presence of bAVMs (*p* < 0.001). Specifically, bAVMs were highly prevalent in ENG mutation carriers (35.8%) compared to ACVRL1 carriers (3.6%). Pulmonary AVMs were also significantly associated with mutations in the ENG gene (*p* < 0.001). Regarding hepatic AVMs, while they were more frequent in ACVRL1 carriers (44.6%) compared to ENG carriers (26.4%), this association did not reach statistical significance (*p* = 0.086).

**Table 2:** Association between mutated gene and presence of major vascular malformations

Phenotype	ACVRL1 *n* (%)	ENG *n* (%)	SMAD4 *n* (%)	*p* value
*Brain AVMs*				< 0.001
Present	3 (3.6%)	19 (35.8%)	1 (16.7%)	
Absent	80 (73.5%)	34 (64.2%)	5 (83.3%)	
*Pulmonary AVMs*				< 0.001
Present	17 (20.5%)	32 (60.4%)	1 (16.7%)	
Absent	66 (79.5%)	21 (39.6%)	5 (83.3%)	
*Hepatic AVMs*				0.086
Present	37 (44.6%)	14 (26.4%)	3 (50%)	
Absent	46 (55.4%)	39 (73.6%)	3 (50%)	

### Genotype and acute cerebrovascular events

We further investigated the correlation between the underlying genotype and the occurrence of acute cerebrovascular manifestations (Table 3). In our cohort, ischemic stroke was reported in 15 patients (10.6%) and intracranial bleeding in 7 (4.9%). Although ENG mutation carriers exhibited a trend toward a higher prevalence of both stroke (17.0% vs. 6.0% in ACVRL1) and intracranial bleeding (9.4% vs. 2.4% in ACVRL1), these differences did not reach statistical significance (*p* = 0.113 and *p* = 0.155, respectively). Furthermore, an analysis of the specific exon/intron location of the variants did not reveal any significant association with the occurrence of stroke (*p* = 0.090).

**Table 3:** Association between genotype and acute cerebrovascular events

Clinical event	ACVRL1 (*n* = 83)	ENG (*n* = 53)	SMAD4 (*n* = 6)	Total cohort (*n* = 142)	p value
*Ischemic stroke*					0.113
Present	5 (6.0%)	9 (17.0%)	1 (16.7%)	15 (10.6%)	
Absent	78 (94.0%)	44 (83.0%)	5 (83.3%)	127 (89.4%)	
*Hemorrhagic stroke*					0.155
Present	2 (2.4%)	5 (9.4%)	0 (0%)	7 (4.9%)	
Absent	81 (97.6%)	48 (90.6%)	6 (100%)	135 (95.1%)	

### Multivariable logistic regression for brain arteriovenous malformations

To identify independent predictors of bAVMs, a multivariable logistic regression model was constructed using bAVM presence as the dependent variable (Table 4). The model included age, sex, mutated gene (with ACVRL1, ENG, and SMAD4 as factors), and Curaçao score. The overall model was statistically significant (*p* < 0.001).

**Table 4:** Results of multivariable logistic regression for prediction of brain AVMs

Predictor	*β* (SE)	Odds ratio (OR)	95% CI for OR	*p* value
Age	− 0.032 (0.016)	0.968	0.939–0.998	0.04*
Curaçao Score	0.572 (0.406)	1.772	0.799–3.931	0.159
Sex (male vs. female)	0.065 (0.531)	1.067	0.377–3.021	0.902
Gene (ACVRL1 vs. other)	− 1.834 (1.313)	0.160	0.012–2.096	0.163
Gene (ENG vs. other)	0.809 (1.208)	2.246	0.210–23.968	0.503

Significant values are reported in bold

*β* beta coefficient, *SE* standard error, *CI* confidence interval

After accounting for other factors, the model identified patient age as the only statistically significant independent predictor (*p* = 0.040). The Odds Ratio (OR) for age was 0.968 (95% CI 0.939–0.998), suggesting a slight decrease in the odds of having a bAVM for every year of increasing age. While the ACVRL1 genotype showed a trend toward protection (OR: 0.160), this did not reach statistical significance in the multivariable context (*p* = 0.163), nor did the ENG genotype (*p* = 0.503).

### Multivariable logistic regression for other vascular phenotypes

To evaluate the specificity of the predictors identified for brain AVMs, the same multivariable logistic regression model (including age, sex, gene, and Curaçao score) was applied to other vascular phenotypes.

Due to the low prevalence of other cerebrovascular lesions, the regression models for cerebral cavernomas, developmental venous anomalies (DVAs), dural arteriovenous fistulas (dAVFs), and intracranial aneurysms (IAs) did not yield statistically significant independent predictors.

For hepatic AVMs, both Age (*p* = 0.029) and Curaçao Score (*p* < 0.001) were significant independent predictors. Notably, the age effect for liver involvement was the inverse of that observed for the brain: older age was significantly associated with a higher likelihood of hepatic AVMs.

For pulmonary AVMs, the Curaçao Score was the sole significant independent predictor (*p* < 0.001) in the multivariate context.

### Impact of mutation type and protein location domain on clinical phenotype

To further explore genotype–phenotype relationships, mutations were stratified by their functional type (truncating vs. non-truncating) and by their location within the protein domain.

The analysis based on mutation type revealed no statistically significant association between truncating mutations and the presence of either brain, pulmonary or hAVMs (Table 5**).**

**Table 5:** Association between mutation type and major clinical manifestations

Phenotype	Truncating variant *n* (%)	Non-truncating variant *n* (%)	*p*-value
*Pulmonary AVMs*			0.6
Present	27 (37.5%)	23 (33%)	
Absent	45 (62.5%)	47 (67%)	
*Brain AVMs*			0.8
Present	11 (15%)	12 (17%)	
Absent	61 (85%)	58 (83%)	
*Hepatic AVMs*			0.9
Present	27 (37.5%)	27 (39%)	
Absent	45 (62.5%)	43 (61%)	

Based on predicted location, 57 mutations (40.1%) affected the extracellular domain, 50 (35.2%) affected the intracellular domain, and 35 (24.6%) were in the untranslated domain. Stratifying mutations by their predicted protein domain location revealed a significant association was found between the protein domain and the presence of both bAVMs (*p* = 0.034) and cavernomas (*p* = 0.020), with mutations in the extracellular domain conferring a higher risk for these cerebrovascular malformations. In contrast, a strong association was identified between mutations in the intracellular domain and pAVMs (*p* = 0.008).

## Discussion

HHT patients often exhibit a wide variety of cerebrovascular diseases. The most common are bAVMs (10–20%), while pial AVFs or capillary vascular malformation are not as frequent [5]. Nonetheless, some studies have also observed associations with other vascular lesions, such as DVAs, IAs, and cavernous malformations [3, 12, 13]. However, the small sample sizes and the selection bias limit the reliability of these findings. Thus, the present study, by analyzing genotype–phenotype correlations in genetically confirmed Caucasian patients with HHT contributes to clarifying those doubts.

### Brain AVMs and aneurysms

Our findings clarify the distribution of cerebrovascular phenotypes in HHT. Consistent with established literature [12, 13], we confirmed the association between ENG variants and pulmonary AVMs (*p* < 0.001). Regarding hepatic AVMs, while ACVRL1 carriers exhibited a higher prevalence (44.6%) compared to ENG carriers (26.4%), this difference represented a statistical trend (*p* = 0.086) rather than a significant correlation in our cohort.

Crucially, regarding cerebral involvement, our data demonstrate a strong, statistically significant association between ENG variants and bAVMs (*p* < 0.001). In our cohort, 35.8% of ENG carriers presented with bAVMs compared to only 3.6% of ACVRL1 carriers. This finding confirm our previous observations regarding the frequency of brain vascular anomalies was significantly higher among ENG carriers (25%) compared with ACVRL1 carriers (13%) [14]. Similarly, Azma et al. [9] reported that in a pediatric cohort, brain AVMs were significantly more frequent among ENG mutation carriers (19.7%) than ACVRL1 (4.9%). Our data reinforce the hypothesis of a genotype-dependent susceptibility where ENG mutations confer a higher risk for high-flow cerebral lesions.

In contrast, the possible association between intracranial aneurysms (IAs) and HHT remains variable in the literature [15]. We observed a 2.8% prevalence of IAs, which aligns with reports by Cheng et al. and Ring et al., suggesting that IAs in HHT patients likely reflect sporadic population rates rather than a specific genetic syndromic feature [1, 7]. Furthermore, unlike bAVMs, we found no significant association between the mutated gene and the aneurysm phenotype.

### Effect of age and multivariable predictors

To further define risk, we performed a multivariable logistic regression. Interestingly, while genotype was the dominant factor in bivariate analysis, patient age emerged as the sole statistically significant independent predictor in the multivariable model (OR: 0.968; *p* = 0.040). This inverse relationship, where younger age is associated with a higher likelihood of bAVM diagnosis, supports the hypothesis that HHT-related brain AVMs are congenital or developmental lesions present from childhood, rather than acquired lesions that accumulate with age.

The independent association between younger age and the presence of bAVMs likely reflects the developmental nature of HHT‑related cerebral vascular malformations, which are generally considered congenital or early life lesions rather than abnormalities that accumulate over time. At the same time, we acknowledge that earlier and more extensive imaging in clinically more symptomatic ENG carriers may contribute to this association, representing a potential source of residual confounding.

This finding stands in distinct contrast to the natural history of intracranial aneurysms in HHT. For instance, Cheng et al. (2023) reported an increased rate of IAs in older individuals. Similarly, Ring et al. (2021) demonstrated that older age and ACVRL1 mutations were associated with a higher risk of systemic arterial aneurysms (OR: 4.0), finding a 4.3% prevalence of IAs compared to 1.8–3.2% in the general population. These converging observations suggest that vascular remodeling evolves differently across the lifespan in HHT patients: bAVMs appear to be developmental anomalies (strongly associated with ENG in bivariate analysis), whereas aneurysms may be acquired lesions driven by hemodynamic stress over time. In our multivariable model, the specific gene (ACVRL1 Vs. ENG) did not retain statistical significance as an independent predictor (*p* = 0.163). This may be attributable to the collinearity between predictors or the sample size of bAVM events relative to the model parameters. However, the strong signal from the age variable is clinically actionable. It supports current guidelines advocating for “screening at diagnosis”, particularly in pediatric and young adult patients, to prevent rupture of these high-flow lesions.

### Risk of cerebrovascular events

Our study also explored the relationship between specific genotypes and acute neurovascular complications. While we observed a higher frequency of stroke in ENG mutation carriers compared to ACVRL1 (17.0% vs. 6.0%), this trend did not achieve statistical significance in our cohort (*p* = 0.113). From a pathophysiological perspective, a higher incidence of stroke in ENG carriers would be consistent with the well-established predominance of pulmonary AVMs in this subgroup, which serves as the primary mechanism for paradoxical embolism. The lack of statistical significance in our analysis likely reflects the multifactorial etiology of stroke and the limited sample size for these specific low-prevalence events, suggesting that larger multicentric cohorts may be required to definitively confirm this risk stratification.

### Other cerebrovascular malformations and comparative risk

DVAs, usually benign and often incidental, are among the most common intracranial vascular anomalies in the general population, with ranges varying between 2.6 and 6.4% [16]. Unlike brain AVMs, DVAs have not shown any clear association with the HHT genotype. In fact, both previous literature and our current analysis show rates representative of sporadic occurrences (4.2%) rather than pathognomonic patterns. Our secondary multivariate analysis confirmed this distinction: we found no independent demographic or genetic predictors for DVAs. This suggests that these lesions do not share the same strict developmental determinants as HHT-related bAVMs and likely represent incidental findings overlapping with the syndromic phenotype.

Regarding cerebral cavernomas, a recent systematic review [2] analyzed patterns of non-AVM cerebrovascular malformations, finding that cavernomas occurred in the HHT population at approximately the same rate as the general population (0.2%). Our findings report a slightly higher rate (2.8%); however, this was not statistically significant, and our regression models similarly identified no specific risk factors for their presence.

The most notable findings appear when evaluating the incidence of dAVFs. Despite not reaching statistical significance, we found a 0.7% rate of dAVFs in our cohort. This aligns with evidence from the Palermo et al. (2025) review, which reported a 1.2% rate of high-flow arteriovenous shunts, exceedingly higher than the estimated prevalence in the general population. However, we acknowledge that this finding does not provide definitive evidence of causality as only 1 patient in our cohort developed a dAVF, and multivariate analysis yielded no independent predictors.

Collectively, the lack of predictive factors for these lesions stands in sharp contrast to our findings for major visceral AVMs. Our secondary analysis provided a crucial insight regarding the natural history of the disease: we found that older age was a significant predictor for Hepatic AVMs (*p* = 0.029), consistent with the known age-dependent penetrance of liver involvement. This diverges significantly from our primary finding that younger age predicts brain AVMs (*p* = 0.040). This divergence underscores that HHT vascular remodeling follows organ-specific temporal patterns: likely congenital and static for the brain, versus progressive and accumulative for the liver.

### Protein-level analysis

Our data suggest that the risk of developing a specific subtype of vascular anomaly extends beyond simple organ tropism. In line with this, protein-domain localization analysis further provides more accurate phenotypic stratification.

Extracellular domains were found to be linked to a higher risk of bAVMs and cavernomas, whereas intracellular domain mutations were associated with PAVMs. Nevertheless, nonsignificant difference emerged between truncating versus non-truncating variants. Such findings support the hypothesis that disruption of ligand-binding or receptor–receptor interaction sites (extracellular domains) may compromise endothelial stability in cerebrovascular territories that are heavily dependent on tight TGF-β/BMP signaling gradients.

### Screening

Genetic mutations that cause HHT affect the TGF-b signaling pathway, which is thought to be involved in vascular remodeling. Defects in HHT-associated genes have been shown to be linked to abnormal angiogenesis [1, 17, 18]. Although the common pathological phenotype associated with HHT is a multi-organs vascular malformation characterized by arteriovenous shunt, the dysregulation of the TGF-b pathway has also been implicated in causing aortic aneurysms in patients with connective tissue diseases, such as Marfan, Loeys–Dietz, and Ehlers–Danlos syndromes [8, 19]. Therefore, it is plausible that HHT-related mutations affect arterial vasculature in a way that also increases the risk of aneurysm or other cerebrovascular malformation development [16, 20, 21].

The relevance of these mechanistic inferences is supported by recent pediatric and adult studies emphasizing the genotypic modulation of cerebrovascular risk.

Finally, the clinical implications of genotype-guided vascular screening extend to therapeutic decision-making. As highlighted by Gaetani et al. (2020), antithrombotic therapy in HHT requires careful individualization due to increased bleeding risk, yet can be safely administered under close multidisciplinary supervision [3]. Understanding the molecular determinants of cerebrovascular fragility could, therefore, improve risk stratification not only for surveillance but also for antithrombotic and interventional management.

## Limitations and strengths

The strengths of the present study include its genetically confirmed cohort, systematic radiologic characterization, and incorporation of a protein domain-based analytical framework. Limitations include its retrospective nature and the absence of longitudinal follow-up to assess the temporal evolution of cerebrovascular lesions. Additionally, the study population was ethnically homogeneous, which limits generalizability. Despite these constraints, our results highlight the potential for integrating molecular topography with phenotypic surveillance to move toward precision neurosurgical and neuroradiological care in HHT.

We acknowledge a potential limitation regarding the use of the Curaçao score as a predictor in our logistic regression models. Since the presence of visceral vascular malformations contributes one point to the Curaçao criteria, there is an inherent risk of incorporation bias, particularly for the hepatic and pulmonary AVM models where the score was found to be significant. However, regarding our primary analysis of brain AVMs, the Curaçao score was not a significant predictor. This suggests that the presence of high-flow cerebral lesions does not necessarily correlate with the severity of the mucocutaneous hemorrhagic phenotype (epistaxis and telangiectasias) that primarily drives the score in many patients. A further limitation is the absence of a standardized longitudinal follow‑up, as imaging and clinical assessments were performed according to clinical need rather than predefined intervals, preventing the calculation of a meaningful mean follow‑up duration or incidence estimates.

In contrast to our previous report published in 2022 [13], which included a smaller patient sample who had available neuroimaging at that time and focused primarily on the association between ENG mutations and brain AVMs, the present study offers a wider and more integrated view of cerebrovascular involvement in HHT patients. In fact, here, we examined a larger cohort with a complete neurological and splanchnic radiological screening along with a genetical confirmation of the disease, allowing a more accurate evaluation of the incidence of cerebrovascular malformations across genotypes and a clearer understanding of how cerebral findings relate to systemic vascular involvement.

Beyond exploring gene–organ associations, this work also incorporates pulmonary, hepatic, and additional cerebrovascular malformations (including cavernomas, DVAs, dAVFs, and aneurysms) into a unified genotype–phenotype framework, providing a more comprehensive picture of vascular risk in HHT. Moreover, the introduction of a multivariable logistic regression model further refines these associations by identifying independent predictors of brain AVMs, and in particular the patient age, rather than providing solely unadjusted comparisons.

Finally, the introduction of a protein domain-level analysis represents a conceptual expansion, suggesting that the topography of a variant within the encoded protein may contribute to an additional discrimination beyond the mutated gene itself. Taken together, these elements substantially expand our previous work and offer new understandings into the molecular and clinical determinants of cerebrovascular risk in HHT.

## Conclusion

In conclusion, our findings reinforce classical gene–organ associations while proposing that protein domain-level variant classification may enhance prediction of cerebrovascular risk. Integration of genetic architecture, patient age, and domain-specific signaling disruption may form the basis for individualized screening algorithms and targeted monitoring in HHT.

## Supplementary Information

Below is the link to the electronic supplementary material.

Supplementary file1 (XLSX 68 KB)

## Data Availability

The datasets used and/or analyzed during the current study are available in the Supplementary Material.

## Abbreviations

HHT: Hereditary Hemorrhagic Telangiectasia
AVM: Arteriovenous malformation
bAVM: Brain arteriovenous malformation
pAVM: Pulmonary arteriovenous malformation
hAVM: Hepatic arteriovenous malformation
DVA: Developmental venous anomaly
dAVF: Dural arteriovenous fistula
IA: Intracranial aneurysm
MRI: Magnetic resonance imaging
CT: Computed tomography
US: Ultrasound
ENG: Endoglin
ACVRL1: Activin A Receptor Type II‑Like 1
SMAD4: SMAD Family Member 4
TGF‑β: Transforming growth factor‑beta
BMP: Bone morphogenetic protein
NGS: Next‑generation sequencing
OR: Odds ratio
CI: Confidence interval
GLMM: Generalized linear mixed model
SD: Standard deviation
